# Is patients’ activities of daily living self-care score in Norwegian home care a proxy for workers standing at work?

**DOI:** 10.1186/s12913-024-10897-1

**Published:** 2024-05-09

**Authors:** Fredrik Klæboe Lohne, Marius Steiro Fimland, Charlotte Lund Rasmussen, Ingeborg Frostad Liaset, Heike Fischer, Skender Redzovic

**Affiliations:** 1 Department of Neuromedicine and Movement Science, Faculty of Medicine and Health Sciences, Norwegian University of Science and Technology, Trondheim, Norway; 2 Unicare Helsefort Rehabilitation Centre, Rissa, Norway; 3 Curtin School of allied Health, Curtin University, Perth, Western Australia 6102 Australia; 4 Trondheim municipality, Bergheim home care service, Postboks 2300 Torgarden, Trondheim, 7004 Norway

**Keywords:** Occupational health, Constrained standing, Health care services, Compositional data analysis, Musculoskeletal pain, Norway

## Abstract

**Background:**

Prolonged standing at work may contribute to increased risk of musculoskeletal pain in home care workers. Patients’ activities of daily living (ADL) score may be a proxy for home care workers’ standing time at work. The objective of the present study was to investigate the association between patients’ ADL self-care score, and workers standing time.

**Methods:**

This cross-sectional study measured time spent standing, sitting and in physical activity for seven days using thigh-worn accelerometers, among 14 home care workers. Patients’ ADL self-care scores are routinely adjusted by home care nurses, and time intervals of home care visits are stored in home care services electronic patient journal. We collected ADL self-care scores and start and end time points of visits, and categorized ADL self-care scores as low (ADL ≤ 2.0), medium (ADL > 2.0 to 3.0) or high (ADL > 3.0). Physical behavior data were transformed to isometric log-ratios and a mixed-effect model was used to investigate differences in physical behavior between the three ADL self-care score categories.

**Results:**

We analyzed 931 patient visits and found that high ADL self-care scores were associated with longer standing times relative to sitting and physical activity, compared to low ADL score (0.457, *p* = 0.001). However, no significant differences in time spent standing were found between high and medium ADL patient visits (0.259, *p* = 0.260), nor medium and low (0.204, *p* = 0.288). High ADL score patients made up 33.4% of the total care time, despite only making up 7.8% of the number of patients.

**Conclusion:**

Our findings suggest that caring for patients with high ADL self-care score requires workers to stand for longer durations and that this group of patients constitute a significant proportion of home care workers’ total work time. The findings of this study can inform interventions to improve musculoskeletal health among home care workers by appropriate planning of patient visits.

**Supplementary Information:**

The online version contains supplementary material available at 10.1186/s12913-024-10897-1.

## Background

Norwegian healthcare is shifting its focus to home care, reserving institutional care for patients who can’t live at home or need urgent medical attention [1]. Among Norwegians aged 80 and older, 92.4% live in private households [2], and home care services are a cost-effective solution for the growing population in need of care [3, 4]. Furthermore, home care facilitates “aging in place”, a concept where the elderly population remains in the community, as opposed to moving into residential care facilities. This approach is often preferred by the older population, as it maintains continuity of their lifestyle and sense of autonomy [5–7]. With the expected rise in the number of elderly individuals requiring home care services to facilitate aging in place in the coming decades [3, 4, 8], the role of home care workers will become increasingly important. However, the home care sector struggles with high sick leave rates, which at 11% is almost double the Norwegian average (6%) [9]. While sick leave is multifactorial, one possible contributor is that home care workers spend on average more than 30% of their workday standing [10]. Prolonged constrained standing is associated with increased risks of low back pain [11], muscle and cardiovascular problems, fatigue, and discomfort [12, 13].

The ability to perform activities of daily living (ADL) serves as an indicator of a person’s functional status. ADLs, including self-care, eating, mobility, and domestic responsibilities are essential to maintain health and independence. Conversely, the inability to perform ADLs strongly predicts institutionalization [14] and mortality [15] and is associated with lower quality of life [16]. Therefore, assessing a person’s ADL capabilities is crucial for evaluating their functional status, and determining their need for assistance. In Norway, the home care system utilizes the World Health Organization’s International Classification of Functioning, Disability and Health (ICF) [17] to regularly evaluate all home care patients [18]. Within the ICF framework, a central ADL domain regarding workers physical workload, is self-care. This domain assesses a person’s ability to maintain their own hygiene, dress, and undress, eat, use the toilet, and move indoors and outdoors. Providing assistance with these activities is an essential task performed by home care workers during their workday [19]. Therefore, caring for patients with higher ADL self-care needs likely requires prolonged standing for workers as they provide required care. In contrast, patients with lower needs may require more dynamic and simpler tasks.

Home care workers spend a large part of the workday (approximately 50%) on direct patient care assignments [20, 21]. Therefore, if patients’ ADL self-care score functions as a proxy of home care workers’ standing time at work, the home care sector has a nationally available score that can be used to distribute the occupational standing time in a more health-promoting manner. One previous study reported a patient rating system for categorizing patients by their physical workload demand [22], but there is little information about using ADL scores to indicate occupational standing demands.

The main aim of this study was to investigate the standing time of home care workers at assignments of different levels of care need, as indicated by the patients’ ADL self-care score. We hypothesized that home care workers spend more time standing relative to sedentary and active behaviors on assignments caring for patients with a high ADL score compared to those with lower ADL scores. A second aim was to describe the characteristics of assignments in terms of time use, to better understand how the different ADL score categories affect the total workday of home care workers.

## Methods

### Study design and setting

This cross-sectional study utilized physical behavior (i.e., sitting, standing, walking, running, stair walking, and cycling) data collected in a pre-to-post design feasibility study. The study tested an intervention aimed at altering the transportation mode used by home care workers. The home care workers’ physical behavior data from one home care unit located in Trondheim, Norway, were combined with ADL data of the patients attended to by those home care workers. Physical behavior data were collected during fall 2021, while ADL data were extracted from electronic patient journal during spring 2022. The inclusion criterion was employment in a home care position involving patient care. Participants were excluded from physical behavior measurements if they had a fever, were pregnant, had < 50% full time equivalent, or were allergic to the tape used to attach the accelerometer.

In Norway, municipalities organize home care services. Depending on municipality size, home care services are divided into separate home care units based on geography. In the current study, the home care unit from which workers were recruited is responsible for patients in a suburban area within the third-largest municipality of Norway, which has 180,000 inhabitants. The study was approved as part of a larger project (#315556) by the Regional Committee for Medical Research Ethics Central Norway (REK central).

### Data collection

#### Questionnaire and anthropometric measures

The participants in the study completed a questionnaire that included items on gender, age, working title, seniority, and a 0–10 score on the Work Ability Index (WAI). The WAI score was used to measure how well the workers were currently able to perform their work, relative to their lifetime best. Height and bodyweight were measured using a wall-mounted height measure band (Seca 206) and a standard digital bathroom scale.

#### Physical behavior measurements

To measure physical behavior, a tri-axial accelerometer (Axivity AX3; Newcastle upon Tyne, UK) was attached to the proximal part of the thigh, 10 cm above the patella, using medical tape (Opsite Flexifix) to ensure that it remained in place and for waterproofness. The accelerometer was set up to record at 25 Hz at ± 8 g for 7 days using the software OmGui.

#### ADL and assignment duration

Assessments of patients’ ADL scores are conducted by home care nurses, and updated twice yearly, where the individual subcategories are evaluated by the current level of functioning. The ADL scores are stored in an electronic patient journal which the home care unit uses to track patients’ health markers and record all relevant information about the patients. In the same electronic patient journal, the start and end time of every assignment is recorded. We define the assignment as the work performed by home care workers between the recorded start time, i.e., when they enter the patient’s home, to end time, i.e., when they exit the patient’s home. Accessing the electronic patient journal allowed us to collect the duration of assignments, along with the respective patient’s ADL self-care score, giving the individual assignments an ADL score. A nurse who was a member of the research team (HF) and had pre-approved access to the home care’s electronic patient journal, collected the data. Using the definitions provided by the Norwegian directorate of health [18, 23], the patients and thus the assignments were categorized as ‘low’, ‘medium’, or ‘high’ based on their ADL self-care score (Table 1).

**Table 1:** Description of how the three ADL score categories were defined

Low ADL self-care score	ADL ≤ 2.0
Medium ADL self-care score	ADL > 2.0 & ≤3.0
High ADL self-care score	ADL > 3.0

ADL: activities of daily living

### Data processing

**Fig. 1:**
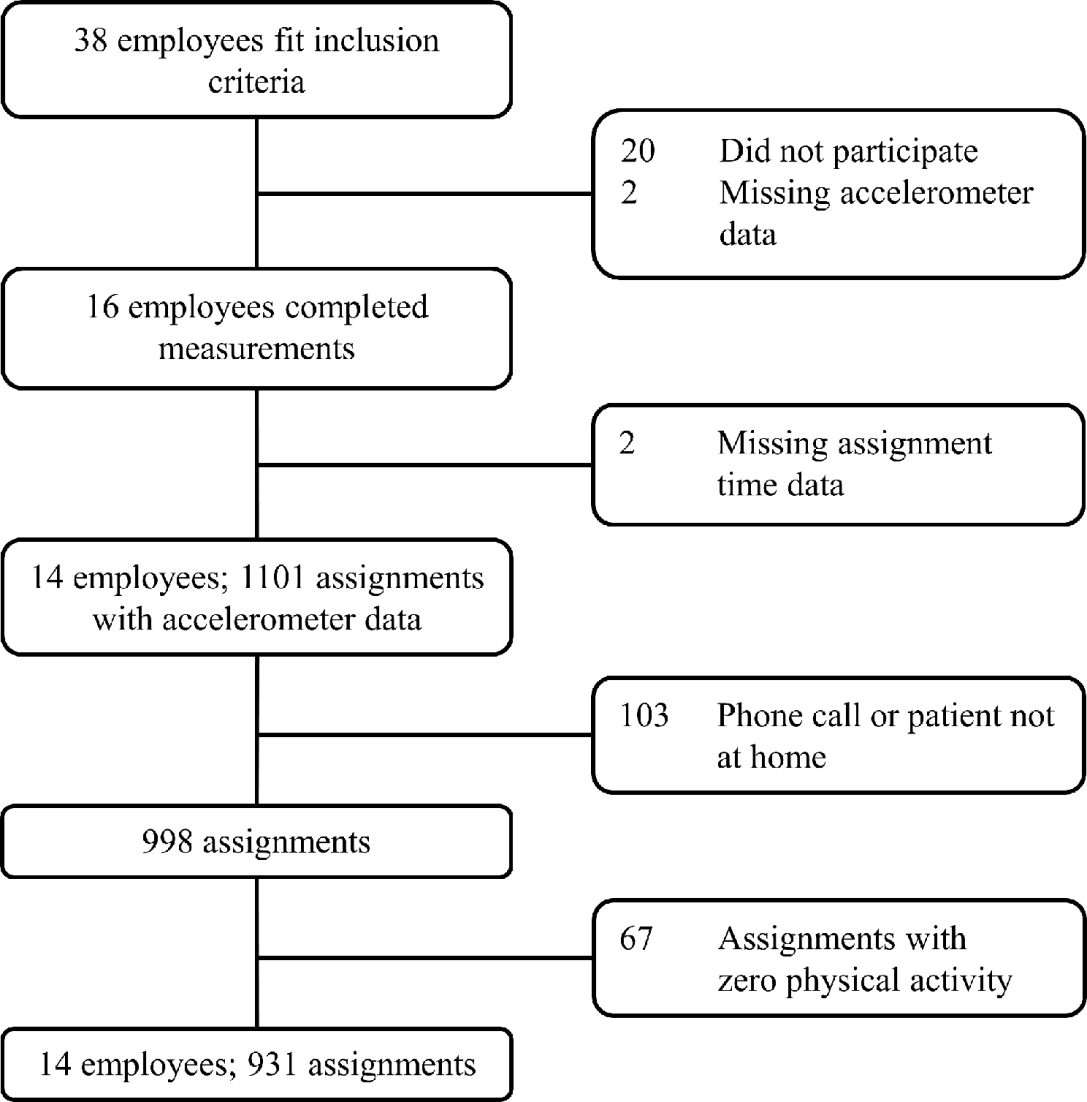
Flowchart for participants and the assignments. Boxes on the right are number of excluded participants/assignments and reason

Accelerometer data were downloaded from the accelerometers and processed using the Acti4 software developed by the National Research Centre for the Working Environment, Copenhagen, Denmark and Department of Work and Health, Federal Institute for Occupational Safety and Health, Berlin, Germany [24]. Acti4 detects the following physical behaviors with high precision: Walking, standing, sitting, running, cycling and stair walking [24, 25]. In this study, the measurement periods were organized into assignments with their respective patients’ ADL self-care score, resulting in a dataset with the amount of time spent in different physical behaviors for each assignment. The time spent in sedentary behavior (sitting), standing (standing still, and standing with small movements), and physical activity (walking, running, stair climbing) for each assignment was then calculated. Assignments that did not involve physical contact with a patient (checking on the patients by phone call or the patient was not at home for a visit) were excluded from the analysis (Fig. 1).

#### Statistical analysis

All data analysis, processing and graphical output were conducted using R (RStudio v. 4.2.1), with the packages “compositions” [26], “zCompositions” [27], and “lme4” [28]. The statistical analysis applied in this study was based on compositional data analysis (CoDA) [29, 30]. The average work time spent in the three physical behaviors—namely, sedentary behavior, standing and physical activity—was conceptualized as a three-part work time-use composition for each assignment. The time-use composition was described using compositional means, which were calculated as the geometric mean for each compositional part. These means were then adjusted to ensure they summed to the median duration for each category of assignments (low, medium, and high), expressed in minutes and totaling 100%. Next, the three-part composition was transformed to isometric log-ratios (ilr). A prerequisite for transforming compositional data to ilr-coordinates is that no part of the composition contains zeros. Thus, multiplicative replacement was conducted before transformation, which requires at least one part to have no zeros for all datapoints i.e., no zeros in either standing, sitting or physical activity [31]. All three physical behavior categories contained zeros, therefore, to have at least one complete physical behavior part, 67 assignments containing zero physical activity were removed, as some walking is inherent in an assignment. Each worker’s time-use composition was then expressed as a set of two ilr coordinates; ilr1 and ilr2 (Eqs. 1 and 2). This way, ilr1 expressed the time spent standing relative to sedentary behavior and physical activity time, while ilr2 expressed time spent sitting relative to the physical activity time.

1 $$ ilr1=\sqrt{\frac{2}{3}}\text{ln}\left(\frac{stand}{\sqrt{sedentary{\ast}physical} \, activity }\right)$$

Equation 1: Formula for calculation of isometric log ratio 1 (ilr1)

2 $$ ilr2=\sqrt{\frac{1}{2}}\text{ln}\left(\frac{sedentary}{Physical \, activity }\right)$$

Equation 2: Formula for calculation of isometric log ratio 2 (ilr2).

Next, to assess whether the differences found in the compositional means between the low, medium, and high ADL score assignments were significant, the relationship between relative standing work time and ADL scores was investigated using compositional mixed effect models. The ADL score was the independent variable with three levels: low, medium, and high, while the ilr-coordinates (ilr1 and ilr2) were modelled as dependent variables. Due to each of the 14 participants having several assignments, participants were used as a random effect. Age and gender were entered as fixed effects as potential confounders. To maintain statistical power, all occupation groups, i.e., nurses, nurse assistants, physiotherapists, occupational therapists, and welfare nurses were included in the same analysis. Tukey adjustment was used as post-hoc adjustment for multiple testing. Alpha was set to 0.05 and residuals vs. fitted plot was checked to assess the model’s goodness of fit.

## Sensitivity analysis

We suspected that the high category, defined as ADL self-care > 3, was not specific enough to distinguish between patients requiring substantial care and those who mostly manage themselves. Therefore, in a sensitivity analysis, we defined an alternative categorization for the ADL scores. The low ADL score category remained unchanged, while the medium was redefined as ADL > 2.0 to < 4.0, and the high category was adjusted to ADL self-care ≥ 4.0. We then conducted an analysis identical to the main analysis using this alternative categorization. In addition, upon discovering some deviations from normality in the residuals vs. fitted plot, we conducted an additional analysis using a robust linear mixed model.

## Results

### Study population characteristics

Out of 38 potential participants at the home care unit, 14 (36.8%) workers participated in physical behavior measurements. Most participants were female nurses working full-time equivalent positions. Nine participants had less than five years of experience in the home care sector. On average, participants rated their ability to work as high at 8.3 (Table 2).

**Table 2:** Descriptive table of participants and workplace included in the study

Variable	N (%)	Mean (SD)
Worker characteristics		
*Total participants*	14 (100)	
*Female*	11 (78.6)	
*Age (years)*		37.3 (13.3)
*BMI (kg/m*^*2*^*)*		24.9 (4.9)
*Work ability index (0–10)*		8.3 (2.0)
Employment		
*Full time equivalent 100%*	10 (71.4)	
*Full time equivalent 60–99%*	4 (28.6)	
*> 10 years working in homecare*	3 (21.4)	
*5–10 years working in homecare*	2 (14.3)	
*< 5 years working in homecare*	9 (64.3)	
Job title and assignments		
*Nurse*	5 (35.7)	
*Assignments*	272 (29.2)	
*Assistant Nurse*	4 (28.6)	
*Assignments*	320 (34.3)	
*Occupational therapist*	2 (14.3)	
*Assignments*	151 (16.2)	
*Welfare nurse*	2 (14.3)	
*Assignments*	126 (13.5)	
*Physiotherapist*	1 (7.1)	
*Assignments*	62 (6.7)	

ADL: Activities of daily living, BMI: Body mass index

The dataset comprised 931 assignments from 14 participants. Patients classified as having high ADL accounted for 20.6% of the total assignments and 33.4% of the total care time, despite comprising only 7.8% of the total patients. The complete breakdown of assignments and patients can be found in Table 3.

**Table 3:** Description of assignments by the different ADL score categories, including both main analysis and sensitivity analysis

	Main analysis	Sensitivity analysis
Total	**N (%)**	**N (%)**
*Total patients*	229 (100)	229 (100)
*Total assignments*	931 (100)	931 (100)
*Total hours*	270.4 (100)	270.4 (100)
Low ADL		
*Low ADL patients*	149 (65.0)	149 (65.0)
*Number of assignments*	411 (44.1)	411 (44.1)
*Total hours*	82.1 (30.4)	82.3 (30.4)
Medium ADL		
*Medium ADL patients*	62 (27.1)	77 (33.6)
*Number of assignments*	326 (35.2)	456 (49.1)
*Total hours*	98.0 (36.2)	155.4 (57.5)
High ADL		
*High ADL patients*	18 (7.8)	3 (1.3)
*Number of assignments*	194 (20.6)	64 (6.6)
*Total hours*	90.3 (33.4)	32.7 (12.1)

ADL = activities of daily living

### Compositional description of assignments

**Fig. 2:**
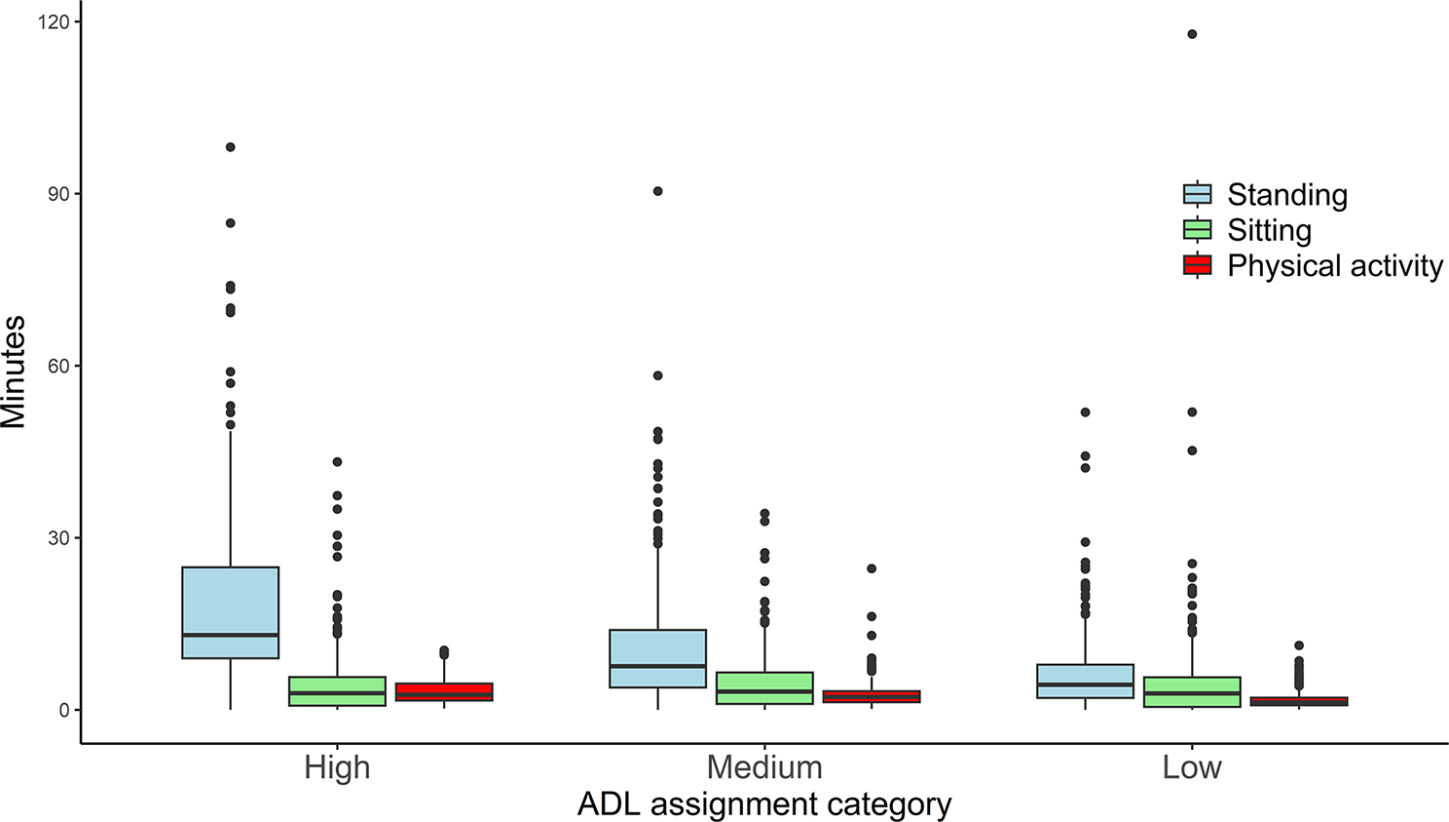
Boxplot showing time use characteristics of the individual assignment separated into high, medium, and low activities of daily living (ADL) categories. Values are in absolute time

Table 4 presents the compositional means of time spent in physical activity, standing, and sitting, normalized to the median duration of the assignments. The analysis revealed that standing was the most prevalent behavior across all assignment types, with high ADL scoring assignments containing relatively more standing time than medium and low ADL scoring assignments. Sitting duration was higher for low ADL scoring assignments compared to medium and high. Figure 2 is a boxplot illustrating the differences between assignments. The boxplot shows a considerable number of outliers indicating a high degree of variation within ADL score categories.

**Table 4:** Description of each type of assignment with regards to how much standing, sitting, and physical activity (walking, running, stairclimbing). Represented by duration normalized to the median duration in minutes of an assignment and the compositional mean of the duration in percentage

	**Description of assignments, main analysis**		
ADL category	High	Medium	Low
Duration, median	21.0 min	14.0 min	10.0 min
Standing (%)	15.6 min (74.4%)	9.1 min (65.0%)	6.1 min (60.4%)
Sitting (%)	2.2 min (10.8%)	2.2 min (16.0%)	1.9 min (18.7%)
Physical activity (%)	3.1 min (14.9%)	2.6 min (18.9%)	2.1 min (20.9%)
	**Description of assignments, sensitivity analysis**		
Total	22.0 min	15.5 min	10.0 min
Standing (%)	16.9 min (76.9%)	10.5 min (67.5%)	6.0 min (60.5%)
Sitting (%)	2.3 min (10.2%)	2.2 min (14.5%)	1.9 min (18.8%)
Physical activity (%)	2.8 min (12.9%)	2.8 min (18.0%)	2.1 min (20.7%)

### Linear mixed models

The mixed model results demonstrated that the difference in time spent standing found between high and low ADL score assignments was significant (0.460, *p* < 0.001). However, there were no significant differences between high and medium ADL score assignments (0.256, *p* = 0.269), nor between medium and low ADL score assignments (0.204, *p* = 0.288). Estimated means and 95% confidence intervals from the mixed linear models can be found in Additional file 1. There were no significant differences in time spent sitting or in physical activity across the different ADL scores.

### Sensitivity analysis

The results were similar when using the alternative categorization of ADL scores, revealing a significant difference between high and low ADL scoring assignments (0.507, *p* = 0.046). However, the higher cutoff for the high ADL category led to a significant difference between medium and low ADL score assignment (0.276, *p* = 0.023). The difference between high and medium remained non-significant (0.230, *p* = 0.777). Further, after observing some deviation from linearity in the fitted vs. residuals plot, a robust mixed-effect linear regression was conducted, which yielded the same results as the main analysis. Given the similar results, the non-robust model was retained.

## Discussion

To our knowledge, this is the first study to investigate the association between levels of patient care needs, as categorized by their ADL self-care score, and workers standing time during home care assignments. As hypothesized, despite taking into account duration differences between assignments, home care workers spent more time standing when caring for patients with higher care needs compared to patients with lower care needs. Although patients with a high ADL self-care score constitute a small percentage of the total amount of patients (7.8%), they accounted for a substantial proportion of the total work time (33.4%) among home care workers.

The results from this study indicate that the ADL self-care score of patients in the Norwegian home care system may function as an indicator for standing time amongst home care workers. However, the boxplot (Fig. 2) showed considerable variation within as well as between categories. Therefore, the specificity of the categories may be low. To determine if an alternative categorization impacts results, we performed a sensitivity analysis with a narrower “high” category. The sensitivity analysis produced similar results to the main analysis regarding the relative standing time, as there was a significant difference between high and low ADL score assignments. However, in contrast to the main analysis, there was a significant difference between medium and low ADL assignments. This difference is likely due to more demanding patients being categorized as medium, resulting in an increased difference to low ADL assignments. Moreover, the larger difference in the sensitivity analysis (0.518) compared to the main analysis (0.457) suggests that patients with ADL scores ≥ 4.0 require more standing than those with ADL scores > 3. There was no significant difference between high and medium for either analysis, but the lack of a significant difference may be due to low statistical power resulting from few high ADL assignments. Due to the small differences between the categorization methods, both can serve as an indicator of how much the worker is required to stand during work.

The home care sector has received limited scientific attention, and cross-national comparisons are challenging due to differences in the operational structures of home care organizations [32]. While previous studies have investigated the physical workloads of caregiving assignments, to our knowledge, none have specifically focused on standing time. Similar to the current study, Väisänen et al. [33] used the case mix index, an indicator of the patients care needs in the Finnish health care system. They investigated the relationship between the case mix index and day-time recovery, as measured by heart rate, and heart rate variability. They found that lower daily mean care needs were associated with more day-time recovery, implying a lower cardiovascular strain on workers. Further, Czuba et al. [34] observed 17 home care workers in the US for a total of 54 h of physical behavior data. They found that lifting/lowering, pushing/pulling and carrying made up 66% of motions performed during care for patients with ADL-5, compared to 21–35% for all other ADL scores. They also found a strong trend for an increased proportion of direct patient care tasks with patients of higher ADL scores. In Denmark, nursing homes have no standardized rating of patients’ care needs, so Jacobsen et al. [22] created and validated a patient rating scale as a proxy for the physical work demands of the workers. They used observational data from 1456 patients to assess the physical work demands for four patient categories: in need of “light”-, “moderate”-, “extensive”- to “complete”- physical assistance. They found a moderate to strong positive correlation between increased care needs of patients and the number of patient handlings during the visit, such as lifting, repositioning and turning patients. Using the same dataset, Kyriakidis et al. [35] found a positive association between the number of patients with a higher need for physical assistance and the number of patient handlings during a worker’s shift, indicating a higher physical workload of the entire shift with an increased number of patients with a high ADL score. The research conducted by Czuba et al. [34], Jacobsen et al. [22], and Kyriakidis et al. [35] align well with the current study, and along with the current study, suggest that increased standing time during assignments, may coincide with an increased number of manual handlings of patients, thus presenting an increased risk of musculoskeletal pain [36, 37]. Viewing the current study in the broader context of the aforementioned studies suggest that ADL self-care scores in Norwegian home care organization may be indicative not only of standing time but also of the frequency of manual patient handling, and cardiovascular strain on home care workers. The needs of patients can potentially lead to adverse effects on workers’ health and may result in increased sick leave, high turnover, and early retirement. Furthermore, previous studies have suggested that arm elevation and trunk inclination could be important factors to investigate in relation to standing time during caregiving as they are associated with increased risk of sick leave [38–40]. It is likely that standing time during caregiving assignments involve frequent arm elevation and/or trunk inclination, especially for patients with high ADL scores, who may be bedridden and require extensive assistance with personal hygiene and movement. Future studies should therefore explore the association between ADL categories and arm elevation and/or trunk inclination.

### Practical implications

The amount of standing time and the large proportion of the workday spent caring for high ADL scoring patients is likely due to workers giving extensive care procedures to immobile patients while standing. In contrast, patients with lower care needs may only require help with smaller tasks such as putting on support stockings or taking medications, which do not require prolonged constrained standing, and instead involves shorter durations of other behaviors. Caring for patients with a high ADL self-care score may increase the risk of sick leave, as a consequence of increased risk of musculoskeletal pain from occupational standing [12, 13]. However, these high ADL scoring patients are an essential part of the working day for the home care sector, thus a re-organization of how home care organizes the distribution of these patients may be appropriate. Previous research has described an uneven distribution of physical work demands in home care in Norway [10]. To avoid the increased risk of sick leave that a high workload provides, a more balanced workload between employees could be the solution [41, 42]. A possible way to balance the workload could be through a re-organization ensuring that when employees working shifts containing an above average number of high ADL assignments, they will subsequently work a shift with fewer than average high ADL assignments, thus balancing the total weekly workload more evenly [41]. Furthermore, the implications of the findings from the current study are very likely to be relevant for nursing homes, as patients in nursing homes, often unable to live at home, are more likely to have a high ADL self-care score.

High ADL assignments, on average, had more time standing compared to medium ADL assignments; however, the difference was not statistically significant. We can therefore not conclude that high and medium ADL self-care assignments have different exposure to standing time, and the physical workload may be similar when working with medium and high scoring ADL self-care patients. Future studies should include more participants and more assignments to conclude whether there is a true difference between high and medium ADL self-care assignments.

### Strengths and limitations

The use of accelerometers allowed a reliable assessment of the work time composition of assignments, which also allowed the use of CoDA in the analysis of time use [30, 43, 44]. Secondly, using mixed models allowed keeping the individual assignments in the analysis, as it permits repeated measures within each participant.

The start and end time of assignments were self-reported by the home care workers throughout the working day, increasing the risk of bias, highlighted by some tasks reportedly requiring no physical activity. Further, there is some uncertainty about how well the ADL self-care score reflects the patients’ level of functioning. The scores are supposed to be updated twice yearly by a nurse from the home care unit, but longer periods between each update can occur, while the functioning of patients may change more rapidly. Further, there is a possibility of reverse causality, as patients the workers experience and report as demanding could be more likely to have their ADL score adjusted higher. While we cannot exclude this possibility, we consider the risk of misclassification and reverse causality to be low. Additional measurements of the workload associated with the assignments including heart rate, psychosocial stressors and torque produced by musculatur would have brought additional nuance, to the measure of standing time. Finally, while the dataset consisted of many assignments, the study population was small and all data came from one home care facility; these two factors may limit the generalizability of the results to other facilities and workers. The low study sample also restricted the possibility of conducting sub-group analysis of occupational titles with adequate statistical power, this should be a priority in the future.

## Conclusions

Using data from 931 home care assignments, our study revealed that providing care to patients with high ADL scores involves a greater amount of standing work time compared to other behaviors, especially when contrasted with patients with low ADL scores. Despite being a minority, patients with high ADL scores demand a substantial amount of work time from home care workers.

Based on these findings, we encourage policymakers, home care service management, and researchers to consider patients’ ADL scores when implementing measures and interventions to mitigate potential adverse effects on the health of home care workers. Nevertheless, to enhance our understanding of the relationship between ADL categories and work demands, further research should be conducted including additional measurements such as arm elevation and trunk inclination, heart rate, psychosocial stressors, and muscle torque. Furthermore, studies with more participants and assignments are necessary to conclusively determine differences between high and medium ADL self-care assignments. Subsequent research should investigate the effective utilization of the ADL scoring system in Norwegian home care interventions, with a focus on designing and implementing interventions that distribute occupational workloads in a manner conducive to health promotion.

### Electronic supplementary material

Below is the link to the electronic supplementary material.

Supplementary Material 1

## Data Availability

The datasets used and/or analysed during the current study are available from the corresponding author upon reasonable request.

## Abbreviations

ICF: International Classification of Functioning, Disability and Health
ADL: Activities of Daily Living
CoDA: Compositional Data Analysis
WAI: Work Ability Index
