# Supplementary material for: Is patients’ activities of daily living self-care score in Norwegian home care a proxy for workers standing at work?
Source: BMC Health Serv Res. 2024 May 9;24:565. doi: 10.1186/s12913-024-10897-1 (PMC11080116; doi:10.1186/s12913-024-10897-1)
Supplement: Supplementary file 1 — Supplementary Material 1 [file 12913_2024_10897_MOESM1_ESM.docx]

**Additional file 1**

Table 1: Estimated marginal means and the 95% confidence interval (95% CI) from mixed-effect models.

|  | Estimated marginal means (95% CI) | | | |
| --- | --- | --- | --- | --- |
| ADL score category | | **High** | **Medium** | **Low** |
|  | | **Main analysis** | | |
| Standing (ilr1) | | *1.354 (1.012; 1.696)* | *1.098 (0.773; 1.424)* | *0.894 (0.575; 1.213)* |
| Sitting (ilr2) | | *-0.209 (-0.579; 0.160)* | *-0.212 (-0.567; 0.143)* | *-0.147 (-0.496; 0.202)* |
|  | | **Sensitivity analysis** | | |
| Standing (ilr1) | | *1.401 (0.975; 1.827)* | *1.171 (0.851; 1.491)* | *0.895 (0.573; 1.216)* |
| Sitting (ilr2) | | *-0.109 (-0.557; 0.338)* | *-0.225 (-0.575; 0.125)* | *-0.093 (-0.484; 0.217)* |

Ilr1: Standing time relative to sitting and physical activity. Ilr 2: sitting time relative to physical activity.
